# Scaling medical-dental integration nationally: outcomes from the MORE Care initiative

**DOI:** 10.3389/fpubh.2026.1753528

**Published:** 2026-02-18

**Authors:** Matthew Vermillion, Christine Kanan, Caroline McLeod, Chelsea Chokas, Renee Clark, Andrea Clark, Danielle Apostolon, Rebekah Mathews

**Affiliations:** CareQuest Institute for Oral Health, Boston, MA, United States

**Keywords:** health equity, interprofessional collaboration, medical-dental integration (MDI), oral health disparities, preventive dental services, primary care integration

## Abstract

Oral health is siloed from broader healthcare systems, contributing to disparities in care. MORE Care™, a Medical-Dental Integration (MDI) model originally launched to address access challenges in rural community health centers and offers a scalable approach to embedding oral health into primary care. This study evaluates its implementation in Ohio, where a two-year pilot engaged nearly 20,000 children across six counties. The program integrated oral health assessments, fluoride varnish, and referral systems into private medical practices, supported by a performance-based payment model. Results showed increases in preventive oral health services and referrals, demonstrating the model's adaptability beyond rural settings. However, challenges in health IT and data sharing highlight the need for continued investment in infrastructure and policy to support whole-person care.

## Introduction

Oral health is an important component of overall health, with documented links to chronic health conditions such as endocarditis, cardiovascular disease, diabetes, and complications during pregnancy and childbirth (1). Despite the established connections between oral and systemic health, oral health is often siloed from other healthcare services with little to no communication or coordination. The division results in gaps in care across the lifespan and, with children, this includes missed opportunities for early and preventive interventions such as dental screenings or fluoride varnish application (2). In the common responsive approach to dental care, patients frequently receive care only when an issue emerges or escalates, increasing the cost and severity of treatment.

These systemic inadequacies are further compounded by persistent inequities in access to care, disproportionately affecting low-income, uninsured, minority and rural populations (3). These populations face challenges that include the inability to pay for care, lack of dental coverage, provider shortages, and cultural barriers (4). Leveraging existing healthcare touchpoints, particularly within primary care settings, can provide innovative ways to address these barriers to oral health care.

Primary care providers can help bridge the gap between medical and dental care. MDI efforts in primary care settings can improve access to oral health care by equipping providers to incorporate basic preventive oral health care (e.g., oral examination and topical fluoride varnish application) in patient visits, a vital role in addressing oral disease (5). Integrating oral health practices and efficient referral mechanisms in the primary care setting helps to foster interprofessional collaboration and handle a broader scope of patient needs (6). To operationalize this potential, oral health integration programs have emerged to support primary care teams incorporating oral health into routine practice, such as CareQuest Institute for Oral Health's Medical Oral Expanded Care (MORE Care^®^).

This study aims to document how MORE Care integrated oral health services into primary care workflows, established closed-loop referral systems between medical and dental providers, tested an alternative payment model to incentivize preventive oral health services, and assessed the feasibility of MDI in rural settings. Desired outcomes included the improvement of oral health access, oral health preventive services, and enhanced care coordination to inform policy recommendations to support person-centered care models inclusive of oral health (7).

## Medical oral expanded care (MORE care)

**Figure 1:**
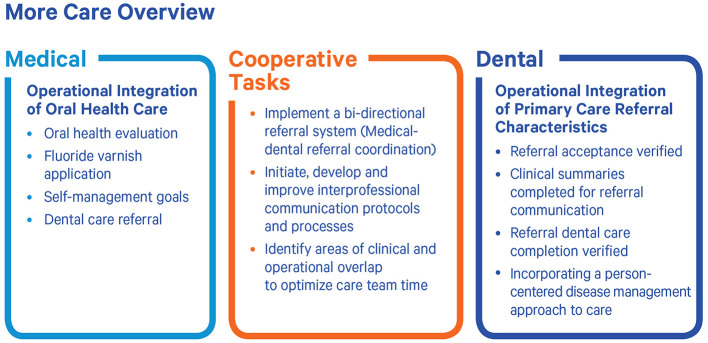
MORE Care Integration Competencies. Source: CareQuest Institute for Oral Health. Partners in progress: pursuing medical-dental integration in Ohio. Boston (MA); 2025 Jul. doi: 10.35565/CQI.2025.2005.

MORE Care is an initiative to help primary care teams develop effective and efficient oral health referral networks, integrate oral health prevention into primary care and develop and test solutions to the challenges of interprofessional practice. Initiative competencies (Figure 1) support those with limited access to care or higher oral disease burden by providing expanded access points and coordination of oral health care (8).

**Figure 2:**
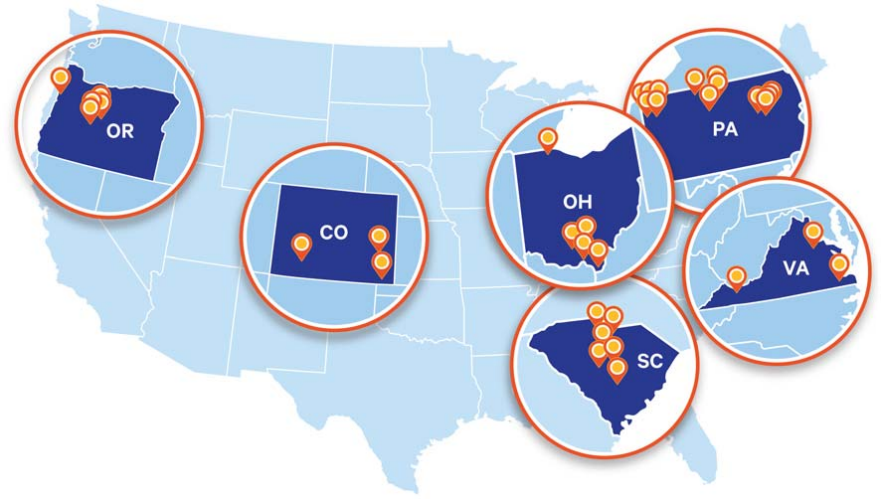
MORE Care Initiative Landscape. Source: CareQuest Institute for Oral Health. Partners in progress: pursuing medical-dental integration in Ohio. Boston (MA); 2025 Jul. doi: 10.35565/CQI.2025.2005

MORE Care began in 2015 by focusing on interprofessional oral health integration within rural primary care clinics in the safety net, but over time it has intentionally expanded into multiple states and care environments (Figure 2). The MORE Care model proved successful for participating CHCs because it provided structured and guided ways to integrate oral health into primary care practice, a gap many health centers struggle with. Across 29 primary care sites in four states, preventive oral health processes improved, with fluoride varnish application from 25 to 40% and documentation of self-management goal documentation increasing from 25 to 62%. Clinics also achieved notable improvements in oral health risk assessment completion, rising from 47 to 77%, despite IT and reimbursement barriers. Qualitative feedback emphasized that the model was particularly effective in CHCs because of the alignment with their mission, patient population, and existing operating model. The practical tools and emphasis on a team-based approach were additional barriers that created sustainable improvements within CHCs (9).

While rural communities face unique barriers such as provider shortages and geographic isolation from oral health care, patients in suburban and urban environments also experience fragmented medical and dental care, with siloed systems that limit early prevention and coordinated treatment. Recognizing this, CareQuest Institute and its partners built on the lessons learned in pilots across rural South Carolina, Pennsylvania, and Colorado to test how the model could adapt to other practice types, including private practice. Through the development of interprofessional networks, integration of workflows into electronic health records, and the creation of standardized referral pathways, the program demonstrated that integration in other settings was not only feasible, but valuable (8). Later phases in Virginia, Oregon, and Ohio showed that adapting MORE Care into pediatric practices and private medical systems could drive measurable improvements in prevention, reduce the need for surgical interventions, and establish stronger links between medicine and dentistry. Most recently, in Ohio, seven private and health-system–affiliated medical and dental achieved a tenfold increase in oral health services during well-child visits, significant growth in preventive dental service delivery, and hundreds of referrals connecting patients to dental care (7). Expanding beyond rural communities is critical, as it demonstrates that the MORE Care framework of integration is scalable, sustainable, and relevant across the full spectrum of care environments. By transitioning from health centers to private practice and health-system settings, the program is demonstrating that integration is not a niche solution for rural access issues but rather a model that can transform oral health delivery nationwide, aligning with broader health system priorities around prevention and population health.

## MORE Care Ohio

MORE Care has been adapted in each iteration since 2015 and tailored to meet each state's unique healthcare landscape, including Ohio. CareQuest Institute and Oral Health Ohio (OHO) partnered to lead the MORE Care Ohio pilot program, advancing MDI and improving pediatric oral health outcomes across the state.

Northwest and Southeast Ohio were identified as key geographic regions for oral health improvement based on oral health access challenges and other social determinants of health. Participants were recruited through targeted outreach to pediatric medical and dental providers in dental Health Professional Shortage Areas with high oral disease prevalence and Medicaid enrollment. Providers were motivated to participate by the opportunities to improve patient outcomes, access training and technical assistance, and receive financial incentives tied to preventive service (7).

By integrating oral health services into pediatric primary care and creating closed-loop referral systems, communication between medical and dental providers improved. From November 2022 to November 2024, the program engaged nearly 20,000 children across six counties by integrating oral health services into pediatric primary care and establishing closed-loop referral systems between medical and dental providers. These efforts were designed to address gaps in access to oral health care, particularly among children covered by Medicaid. The children impacted by the program represented a diverse range of demographic characteristics including gender, age, race, and ethnicity, with detailed characteristics reported previously (7).

This initiative builds on OHO's earlier work convening diverse stakeholders—including public health and private providers, payers, regulators, and advocates—through a series of listening sessions held between October 2020 and August 2021. These sessions explored the future of value-based care (VBC) in dentistry, identified opportunities and challenges, and helped shape a stakeholder-driven model focused on pediatric populations, care delivery, and MDI. The consensus from these sessions emphasized the need for VBC, specifically stronger interprofessional collaboration and sustainable payment models to support preventive oral health services. As one of several managed care organizations operating in Ohio, CareSource played a key role in supporting the MORE Care Ohio pilot program. The organization actively participated in all stakeholder listening sessions that informed the development of the MDI model, contributed insights from a payer perspective and reinforced the importance of aligning oral health with broader health system goals. CareSource also requested and received the Ohio Department of Medicaid's approval to recognize MORE Care Ohio as a Health Care Quality Improvement Activity. This designation signals the initiative meets federal standards set by The Centers for Medicare and Medicaid Services (CMS) for advancing care quality, promoting evidence-based practices, and achieving better health results (10).

To support implementation, MORE Care Ohio used a train-the-trainer model in which CareQuest Institute trained a local Ohio-based implementation coach to work with participants on operationalizing core program components centered on integration. The implementation coach equipped medical and dental providers with the tools, training, and support needed to integrate oral health into their practices and communities. The program paired medical and dental practices and guided them through a structured, phased curriculum that included *Smiles for Life* and MORE Care learning modules, pre-and post-work activities, monthly action calls, and peer-to-peer engagement. MORE Care emphasizes sustainability and the ability to spread successful practices through testing small changes in participant clinical workflows, adopting what worked well, and ensuring these integration efforts continue independently of the program. Primary care practices implemented oral health risk assessments, fluoride varnish applications, oral health self-management goals, and dental referrals during well-child visits. Dental practices focused on preventive care strategies such as caries risk assessment, fluoride varnish, and self-management goals while monitoring patients' need for surgical dental services (ex. tooth extractions, fillings, crowns, root canals). Participants also received personalized coaching and technical assistance to help navigate challenges such as electronic health record limitations, documentation workflows, and referral tracking.

As part of testing the integrated VBC, Ohio stakeholders agreed that the pilot should include a financial incentive structure to promote this prevention-forward approach, which led to the development and implementation of an alternative payment model (APM) to address the limitations of the traditional fee-for-service (FFS) system, which often rewards the volume of services and prioritizes the treatment of oral disease, particularly surgical procedures, over preventive care. In contrast, the MORE Care Ohio APM was structured so participants received their standard FFS reimbursement plus a bonus incentive payment for meeting program performance measure benchmarks (Table 1) tied to preventive services and dental referrals. The APM also included bonus incentive payments for data quality and completeness, encouraging participants to report timely, accurate, and comprehensive data (Table 1) that were used to monitor progress of integration efforts. Importantly, the APM was designed with no downside financial risk, allowing providers to earn financial incentives without penalty if quality measure benchmarks were not met. CareQuest Institute and CareSource provided funding to support incentive payments for participating providers. OHO, serving as the fiscal agent, distributed these payments to the participants periodically as performance was evaluated. CareSource's support highlights how managed care organizations can serve as key partners in advancing health equity and care coordination across traditionally siloed systems.

**Table 1:** MORE Care Ohio quality measures and benchmarks.

**Dental practice performance measures**	**Benchmark**
1. Percentage of patients with caries risk assessment	5% above average baseline count
**2**. Percentage of primary and secondary preventive (dental) procedures	5% above average baseline count
**3**. Percentage of surgical (dental) procedures	5% below average baseline count
**4**. Percentage of patients referred with a dental consultation	Not standard; flat fee rewarded for each referral completed
**Medical Practice Performance Measures**	
**1**. Percentage of patient encounters with an oral health risk assessment	10% of well-child visits
**2**. Percentage of patient encounters with oral health self-management goals	10% of well-child visits
**3**. Percentage of patient encounters with fluoride varnish application	10% of well-child visits
**4**. Percentage of patients referred to a (program) dentist	Not standard; flat fee rewarded for each referral sent
**5**. Percentage of patients referred with a dental consultation and treatment plan received from the dental provider	Not standard; flat fee rewarded for each referral completed
**Data Reporting Measures**	
**1**. Completeness of reported clinical data	
**2**. Completeness of reported demographic and social data	
**3**. Quality of reported data	

Participants achieved the benchmarks for most program months and earned incentive dollars supporting program aims for an overall increase in preventive services and referrals and decrease in surgical dental services compared to pre-program levels, as previously reported (7). The integration of an APM into MORE Care demonstrated that financial incentives can effectively drive improvements in care coordination and encourage provider behavior change. By rewarding the delivery of preventive services and the achievement of performance benchmarks, the APM helped shift participant focus from reactive treatment to proactive, prevention-oriented care. However, it also revealed that financial incentives alone are not sufficient to overcome systemic barriers—particularly technological limitations. A key learning from the Ohio program was that lack of technical infrastructure for primary care and dental practices to report data and securely share referral information necessitated manual referral tracking and reporting processes that, at times, lacked efficiency and data quality. Overcoming these obstacles requires a focus on medical and dental interoperability to fully realize the benefits of integrated care.

## Discussion

MORE Care has evolved with each iteration. A key lesson from the program's evolution is that MDI in primary care is a solution that goes beyond the rural setting and represents a scalable model for oral health care transformation across various care settings. Addressing facilitators and challenges to MDI implementation, including health information technology, payment models, and service delivery will be critical to scalability.

## Interoperability and health information technology

In the context of oral and public health, interoperability refers to the seamless exchange and use of health information across diverse systems, providers, and care settings (11–13). Enabling medical and dental professionals to access, share, and act on patient data in real time supports coordinated care and improves health outcomes.

The current challenges to interoperability are deeply rooted in structural fragmentation, as dental and medical records are typically housed in separate electronic health record (EHR) systems with limited integration. This lack of integration hinders the development of standardized referral pathways, and providers rely on manual processes that are inefficient and prone to error (14). The lack of shared data systems means that even when a referral is made, there is no guarantee it will be tracked, completed, or followed up on. Providers are left without visibility into whether patients received the recommended care, undermining accountability and outcomes. Limitations with health information technology and lack of interoperability have consistently impacted MORE Care since its early days where implementation focused on improving oral health access for underserved populations in rural settings. Coordinated patient care was made difficult by disconnected EHR systems that made data sharing difficult and also impacted workflow efficiency, data accuracy, referral tracking, and reimbursement processes.

MORE Care Ohio demonstrated the value of closed-loop referral systems enabling dental providers to receive and respond to referrals. Although the initiative achieved measurable improvements in preventive care delivery, which included strong stakeholder engagement and financial incentives through an APM, the pilot revealed that technical infrastructure remains a limiting factor. Many practices lacked the capacity to report data electronically or share referral information across systems, requiring manual workarounds that strained staff and compromised data quality.

To address these challenges, policy and infrastructure investments are needed at both federal and state levels. Federal and state agencies like the US Department of Health and Human Services Office of the Assistant Secretary for Technology Policy/Office of the National Coordinator for Health IT (ASTP/ONC) (15) and state Medicaid agencies or insurance departments.

In addition to policy changes, effective strategies can accelerate progress toward interoperability at the provider level. Through the adoption of referral management platforms that integrate with existing EHRs (16), training providers on documentation standards, and incentivizing data sharing can improve care (17) and accelerate progress toward interoperability. Oral health must be embedded into broader health IT strategies, not as an afterthought, but as a core component of whole-person care (16).

Ultimately, these efforts must be guided by a commitment to utilize interoperability as a powerful tool to streamline and structure information exchange processes, which can better ensure that every patient, regardless of where they enter the health care system, experiences coordinated, whole-person care. Whether a patient is seen by a pediatrician, a community health worker, or a dental hygienist, interoperable systems make care more visible and coordinated. In doing so, it advances our main theme: that transforming health requires dismantling silos and creating systems that work for everyone.

## Payment models and incentives for prevention

**Figure 3:**
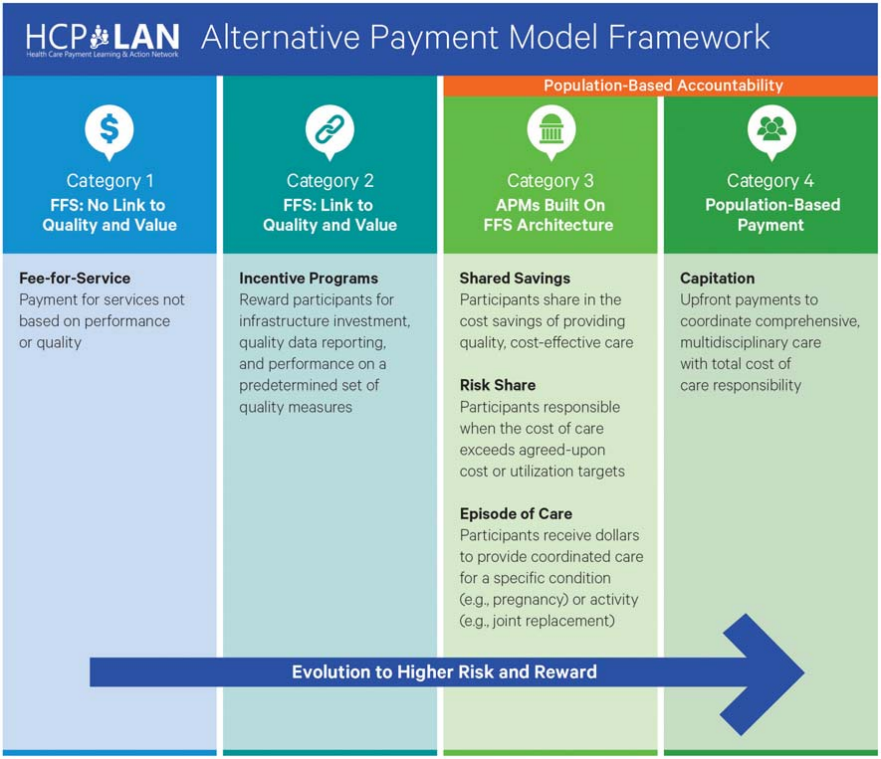
HCP-LAN APM Framework. Source: Health Care Payment Learning & Action Network (HCP-LAN). About us [Internet]. 2023 [cited 2025 Sep 18]. Available from: https://hcp-lan.org/about-us/

For rural primary care providers that participated in earlier MORE Care initiatives, incentives helped to stabilize and offset costs associated with quality improvement work. As the initiative expanded to private practices and larger health systems, more modern APM incentive designs were explored to support MDI. The Health Care Payment Learning and Action Network (HCP-LAN), a national stakeholder group working to accelerate the United States healthcare system's transition to value-based reimbursement through APMs (18) has developed the framework shown in Figure 3 below to illustrate the pathway from volume-based to value- and quality-based reimbursement. MORE Care Ohio tested a FFS + incentive APM model (19).

During the program, MORE Care Ohio's APM provided infrastructural payments (2A), pay-for-reporting incentives (2B) and pay-for-performance incentives (2C) to participants. Both medical and dental practice participants were otherwise reimbursed for service under their normal contractual terms with insurers and patients. Referrals were an intentional part of the incentive design and pay-for-performance models aimed to drive care coordination. While financial motivation encouraged providers to make referrals, technology limitations still posed significant challenges. Some participants lacked critical health information technology to make and complete referrals to medical or dental partners which restricted data capture and reporting capabilities. These gaps highlight a fundamental challenge for leveraging APMs to promote care coordination and interprofessional communication.

Pay-for-reporting and pay-for-performance models such as those piloted during MORE Care Ohio with a pediatric population represent a foundation on which to build value-based reimbursement inclusive of oral health for children and adults. Providers need training and practice to successfully report, measure, and track service utilization (such as the measures listed in Table 1), that provide the basis for determining APM payments. Pay-for-reporting models reimburse providers for the time and resources needed to develop the capability to track and report the data that support successful APM participation. A Level 2 model was also uniquely suited to MORE Care Ohio because the pay-for-reporting and pay-for-performance approaches did not utilize downside risk^1^. These elements of reporting and performance tracking are foundational to Category 3 and 4 HCP-LAN models, which have a stronger emphasis on outcomes. The Category 2 model tested in Ohio prepares dentistry to participate with medical colleagues in collaborative care and payment models. This construct could be explored to promote MDI's spread and sustainability, even if medical practices participate in a variety of contractual arrangements with payers (and potentially higher-level APMs).

MORE Care Ohio shows that payment incentives are not only beneficial as support mechanisms, but can offer a broader solution to align provider, payer and patient interests. The program's insights build on the successes of similar initiatives focused on pediatric populations such as Liberty Dental Plan's B. R. U. S. H. Caries Management program, which provided incentives to Medicaid providers when patient caries decreased or remained low (20, 21), and AmeriHealth Caritas DC's PerformPlus Value Based Compensation Program, where providers are eligible for a per-member-per-month incentive bonus payment based on their performance on select dental and medical utilization specific measures (22). Although the MORE Care and other dental-specific APMs have primarily focused on improving children's oral health, their structures and lessons learned should also inform APMs for adult oral health to advance comprehensive, integrated care across all ages.

## Service delivery lessons from the community health center model

Many Community Health Centers (CHCs) who participated in MORE Care acted as leaders in MDI, offering valuable lessons for private practice dentists and primary care physicians who want to move toward more coordinated, patient-centered care. While CHCs often operate with multidisciplinary teams and built-in links between primary care and dental services, private practices—especially solo or small group offices—function in a very different environment. They don't typically share space with medical and or dental providers and are not federally funded. Still, CHCs' track record shows what is possible, and private practices can adopt many of their successful strategies.

Learnings from CHCs have agnostic application. Private medical practices can follow the same steps to integrate oral health services, like oral health risk assessments, fluoride varnish application and referral protocols, directly into primary care workflows. Private practices can replicate these approaches by adopting tools such as the *Smiles for Life* curriculum, which includes EHR templates, screening checklists, and patient education materials (23). Even without onsite dental colleagues, medical providers can proactively screen, document, and refer patients for dental care. Using an evidence-based approach like this is designed to be efficient, minimize provider burden, and maximize preventive impact.

Referral networks are another area where CHC experience is directly relevant. CHCs benefit from co-located services, but even rural CHCs have proven that integration is possible through structured referral networks. MORE Care Ohio enabled providers to build referral loops with local partners and track patient follow-up (8). Similarly, private practices can begin with low-cost strategies—such as a standardized referral form and closed-loop communication protocols—to ensure patients don't fall through the cracks. Integration via mobile units or telehealth, as demonstrated in CHC participants in MORE Care Colorado and South Carolina, also provides replicable models for overcoming workforce and geographic barriers. These examples show that even a solo physician or dentist can start small—by piloting a referral form with another provider or creating a simple feedback loop—and then scale gradually. The key is building relationships and ensuring patients experience seamless transitions across providers.

Perhaps one of the most influential but underrecognized factors in integration is organizational culture. In CHCs, leadership often fosters a culture that values interprofessional collaboration, shared mission, and patient-centered care. Routine practices such as team huddles, joint trainings, and shared quality metrics promote cross-disciplinary relationships and clarify roles (8).

Conversely, private practices operating in high-productivity, siloed environments which may inadvertently discourage collaboration. However, even without formal infrastructure, private practices can create a culture of collaboration by dedicating time for cross-provider communication, aligning around shared patient goals, and promoting mutual respect across disciplines.

Financial sustainability is often the greatest concern for private practices; however, CHCs offer guidance. CHCs have long participated in value-based payment models that reward prevention, care coordination, and improved outcomes (24). Private practices can also begin to engage in APMs through partnerships with public and private payers, seeking opportunities to enhance reimbursement for preventive services. CHC resources, such as the ASTDD Oral Health and Primary Care Integration Toolkit and the NNOHA Alternative Payment Models Toolkit, provide guidance that private practices can use regarding billing codes, documentation, and payer engagement (25, 26).

While challenges for MDI exist in both CHCs and private practices, it can still be successfully implemented. Adapting CHC-based strategies to workflow design, referral management, interprofessional collaboration, and payment transformation, private practice providers can lead local MDI efforts to build more coordinated and equitable health systems.

## Acknowledgment of conceptual or methodological constraints:

While MORE Care offers important lessons in advancing whole-person care through MDI, several limitations and constraints exist in the findings.

One of the primary challenges for participating practices was the lack of interoperable EHR systems to communicate bidirectionally between medical and dental providers. The limited data sharing led to manual tracking and reporting of referrals, which made the process more difficult to coordinate care while also maintaining accurate and timely data.

While MORE Care has successfully been adopted in CHCs and private practice locations, the variety of practice types has brought differences in staffing models, payer relationships, and workflow structures, influencing how integration strategies were adopted and sustained. Smaller practices were often more agile in adapting workflows and integrating oral health services, while larger, multisite practices faced administrative hurdles and slower adoption.

The APM used in MORE Care Ohio encouraged preventive care but had some evaluation limitations. The evaluation lacked detailed analysis of cost-effectiveness and because providers weren't at financial risk, it was hard to assess how they might behave under more advanced value-based models. Future versions should enhance the APM design to utilize more robust evaluation methods to understand cost and health outcomes.

Demographic data collection was a challenge. Although some demographic data was gathered, gaps remained due to inconsistent software capabilities and the lack of standard procedures for collecting patient information. This made it difficult to evaluate health equity. For example, only medical (and not dental) practices reported data on race and ethnicity. Social drivers of health were not consistently tracked, and without this information, it was hard to evaluate how well interventions addressed oral health disparities.

Finally, while MORE Care showed improvement in preventive care and care coordination, it also demonstrated that financial incentives alone were not enough to overcome systemic barriers. Addressing issues such as technical limitations, policy alignment, staffing constraints, and siloed workflows is crucial to overcoming integration challenges.

## Data Availability

The original contributions presented in the study are included in the article/supplementary material, further inquiries can be directed to the corresponding author.

## Footnotes

1 APMs with downside risk monetarily penalize providers that fail to meet APM benchmarks.
